# Effect of prolonged vibration to synergistic and antagonistic muscles on the rectus femoris activation during multi-joint exercises

**DOI:** 10.1007/s00421-017-3702-1

**Published:** 2017-08-28

**Authors:** Ryoichi Ema, Hirokazu Takayama, Naokazu Miyamoto, Ryota Akagi

**Affiliations:** 1 Graduate School of Engineering and Science, Shibaura Institute of Technology, 307 Fukasaku, Minuma-ku, Saitama-shi, Saitama 337-8570 Japan; 2 Research Fellow of Japan Society for the Promotion of Science, 5-3-1 Kojimachi, Chiyoda-ku, Tokyo, 102-0083 Japan; 3 College of Systems Engineering and Science, Shibaura Institute of Technology, 307 Fukasaku, Minuma-ku, Saitama-shi, Saitama 337-8570 Japan; 4 National Institute of Fitness and Sports in Kanoya, 1 Shiromizu, Kanoya, Kagoshima 891-2393 Japan

**Keywords:** Squat, Electromyography, Reciprocal Ia inhibition, Quadriceps femoris, Vastus lateralis, Biceps femoris

## Abstract

**Purpose:**

Unique neuromuscular activation of the quadriceps femoris is observed during multi-joint leg extensions: lower activation of the biarticular rectus femoris (RF) than monoarticular vasti muscles. As one of the potential mechanisms for the lower RF activation, Ia afferent-mediated inhibitory connections between synergistic muscles and/or between agonist and antagonist muscles have been proposed. If this is the major factor, it is hypothesized that RF activation during multi-joint leg extensions increases after prolonged vibration to synergistic and/or antagonist muscles. This study tested the hypothesis.

**Methods:**

Fourteen men exerted maximal voluntary isometric knee extension and flexion and performed submaximal parallel squat before and after one of the following three interventions on different days: prolonged vibration to the vastus lateralis (VL, synergist) or biceps femoris (BF, antagonist), or quiet sitting for 30 min. Muscle activations of the quadriceps femoris and hamstrings were determined using surface electromyography.

**Results:**

After prolonged VL or BF vibration, VL (21%) or BF (30%) activation during isometric contractions significantly decreased, which was significantly correlated with the reduction of the maximal isometric knee extension or flexion strength. The magnitude of RF activation during squat was significantly lower than those of VL and the vastus medialis. No significant increase in RF activation during squat was observed after vibrations.

**Conclusion:**

The findings suggest that lower biarticular RF activation compared with the monoarticular vasti muscles during multi-joint exercises does not result from the modulation by peripheral inhibitory input from Ia afferents originating from synergist and/or antagonist muscles.

## Introduction

A synergistic muscle group is generally composed of monoarticular and biarticular muscles. Despite their similar functional capability based on anatomical point of view, activation patterns of monoarticular and biarticular muscles have been reported to depend on exercise modality. For example, the quadriceps femoris, which is one of the major contributors to exercise performance (Thorpe et al. 1998), consists of the monoarticular vasti muscles (vastus lateralis, VL; vastus medialis, VM; vastus intermedius) and the biarticular rectus femoris muscle (RF). Some studies showed that the activation of RF during squat (Escamilla et al. 1998, 2001; Ploutz-Snyder et al. 1995) and leg press (Ema et al. 2016a; Escamilla et al. 2001) [multi-joint leg extensions (simultaneous extensions of knee and hip joints)] was lower than those of the vasti at relatively high intensity such as 12-repetition maximum (12 RM) load (Escamilla et al. 1998) and 80% of 1 RM load (Ema et al. 2016a). In contrast, no corresponding difference was found during single-joint knee extensions at high intensity (e.g., 80% of 1 RM load, Ema et al. 2016a). Because RF contraction produces hip flexion torque as well as knee extension torque, and because muscle shortening of RF during multi-joint leg extensions can be smaller than those of the vasti and hence higher force-generating capability (Gregoire et al. 1984), it is possible that less excitatory and/or inhibitory mechanisms exist for RF activation during multi-joint leg extensions. As one of such mechanisms, Ia afferent-mediated inhibitory connections between synergistic muscles (Gritti and Schieppati 1989) and/or between agonist and antagonist muscles (Crone et al. 1987) have been proposed (Ema et al. 2016a; Yamashita 1988). However, these possibilities have not been substantiated by experimental data. Clarifying the issue would improve the understanding of human movement mechanisms, specifically neuromuscular control of the biarticular RF during multi-joint exercises. Moreover, the magnitude of muscle activation during high-intensity resistance exercise was associated with the magnitude of subsequent increase in muscle size (Wakahara et al. 2013), and a high-intensity multi-joint squat exercise increased the vasti muscles’ but not RF sizes (Earp et al. 2015). Thus, focusing on the underpinning mechanisms for the lower RF activation compared with the vasti during multi-joint leg extensions at high intensity is expected to provide significant information regarding the specificity in training response of the quadriceps femoris induced by multi-joint exercises.

A potential way to examine the above possible mechanisms is prolonged vibration. Prolonged vibration to a muscle or tendon substantially affects neuromuscular function. For example, prolonged vibration to the muscle belly of RF for 30 min decreased maximal voluntary isometric contraction (MVC) force of the knee extensors (MVC_KE_) and RF activation during MVC (Kouzaki et al. 2000). The suppressions of the strength and muscle activation are attributable to vibration-induced reduction of Ia afferent fiber activity of the vibrated muscle (Shinohara 2005). Inhibitory connections between synergistic muscles (Gritti and Schieppati 1989) and between agonist and antagonist muscles (Katz et al. 1991) were diminished after prolonged vibration. Therefore, it can be assumed that if the biarticular RF activation during high-intensity multi-joint leg extensions is modulated by synergistic and/or antagonist muscles through Ia afferent-mediated inhibitory connections, RF activation during high-intensity multi-joint leg extensions increases after prolonged vibration to synergistic and/or antagonist muscles because of the diminished inhibitory input from them to RF. This study aimed to examine whether the inhibitory connections is the major factor that regulates RF activation during multi-joint leg extensions through testing the hypothesis.

## Methods

### Experimental design and participants

The participants visited our laboratory on four separate days. On the first day, measurement of the load of 10 RM of parallel squat and familiarization with the strength measurement and squat exercises were conducted. On the second to fourth days, the participants joined the following three interventions during quiet sitting for 30 min in random order: tonic vibration to the right thigh’s VL muscle belly (VL condition), tonic vibration to the right thigh’s biceps femoris long head (BF) muscle belly (BF condition) and quiet sitting without any vibration (CON condition). We selected VL and BF as target muscles since they have relatively large physiological cross-sectional areas (Ward et al. 2009) and muscle volumes (Ema et al. 2016b) among the constituents of the quadriceps femoris and hamstrings, respectively. Before and after the interventions, measurements of maximal isometric knee extension and flexion strength and parallel squat at 10 RM were performed. All measurements were completed within 5 min after the intervention, so the reduction of Ia afferents activity following prolonged vibration for 30 min would still be in effect (Thompson and Bélanger 2002). We performed a priori sample size estimation (G*Power 3.1.7, Kiel University, Germany) to detect a significant change in knee extension torque following VL vibration for 30 min by a paired *t* test, using *α* = 0.05, power at 0.80, and data of our pilot study [*n* = 11, mean of difference and standard deviation (SD) of difference in MVC_KE_ torque before and after the vibration were 13 and 10 Nm, respectively]. The estimation demonstrated that six participants would be needed to find the expected change. In the current study, fourteen untrained healthy men (age, 22 ± 2 year; height, 1.72 ± 0.04 m; body mass, 64 ± 6 kg; mean ± SD) who had no injuries of the lower extremity participated. Prior to the execution of the experiments, the participants were informed of the purpose and risks of the study and provided written informed consent. This study was approved by the Ethics Committee of the Shibaura Institute of Technology.

### Electromyography (EMG) measurements

Surface EMG signals were recorded from VL, VM, RF, BF and semimembranosus (SM) using Ag/AgCl electrodes (BlueSensor N-00-S, Ambu A/S, Denmark) with an interelectrode distance of 20 mm. The electrodes were placed at the level of 90% (VM), 70% (SM), 50% (VL and BF) and 40% (RF) of the thigh length which was determined as the distance from greater trochanter to popliteal crease, after the identification of muscle belly and fascicle directions using B-mode ultrasonography (ACUSON S2000, Siemens Medical Solutions, USA) so as to reduce the effect of cross talk. The electrode placement was preceded by abrasion of the skin surface to reduce the source impedance to less than 5 kΩ. The EMG signals were high-pass filtered (5 Hz) and amplified (MEG-6108, Nihon Koden, Japan). The reference electrode was placed on the right patella for all EMG measurements. To match the electrode placement among the three different conditions, the participants were requested to maintain some pen marks that indicated the electrode placements on the skin throughout the experiments.

### Vibration

The participant sat in a specially customized dynamometer (Hamano Seisakusho, Japan) and remained relaxed during the interventions. The knee and hip joint angles were 75° and 80° (anatomical position = 0˚), respectively. In the vibration conditions, tonic vibration was applied for 30 min perpendicular to right VL (i.e., from right side of the thigh) and BF (i.e., from back side of the thigh) slightly proximal top the region of EMG electrodes using a vibration generator (WaveMaker05, Asahi Seisakusyo, Japan). To selectively activate Ia afferents, the vibration frequency was set at 80 Hz (Roll et al. 1989). The force of the vibration was measured using a load cell (LUR-A50NSA1, Kyowa, Japan) attached to the vibration generator. The forces before and during the vibration and peak-to-peak amplitude of the vibration were controlled at 7 N, 10–15 N and 1.6 mm, respectively. They were similar to those of the previous study that indicated a significant reduction of Ia afferent activity accompanied by the corresponding decrease in MVC torque and agonist muscle activations after 30 min vibration (Ushiyama et al. 2005).

### Strength measurements

**Fig. 1:**
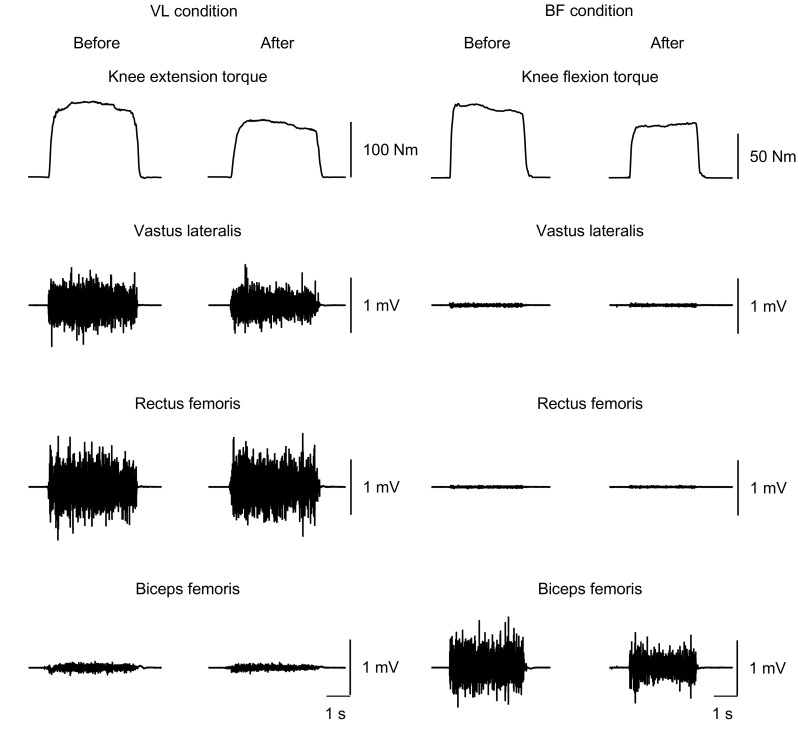
Examples of maximal voluntary isometric contraction torque and electromyographic signals of the vastus lateralis, rectus femoris and biceps femoris long head before and after prolonged vibration. VL condition, tonic vibration to the vastus lateralis during quiet sitting for 30 min; BF condition, tonic vibration to the biceps femoris long head during quiet sitting for 30 min

Before and after the intervention for 30 min, isometric knee extension and flexion strengths with maximal effort were measured (Fig. 1). The participant sat on the bench of the dynamometer with their pelvis secured to the bench by a non-elastic strap. Care was taken to adjust the centers of rotation of the dynamometer and knee joint. The knee and hip joint angles were consistent with those during tonic vibration. Before each intervention, the participant was asked to extend or flex the knee twice with maximal effort. If the difference in peak value between the two contractions was above 10%, a third trial was allowed. Immediately after the 30 min intervention, the knee extension and flexion strength trials were again performed once each. The peak torque was defined as MVC torque (knee extension, MVC_KE_; knee flexion, MVC_KF_).

### Squat exercise

The free-weight parallel squat was performed before and after the intervention. To match the joint kinematics before and after the intervention and among the three conditions, following procedures were performed. The participant was instructed to stand equally on both legs on the floor with their feet shoulder-width apart and angled outward at approximately 30°. The standing position relative to the experimental setup was fixed in each participant throughout the experiment. From the standing posture, the participant performed parallel squats at 10 RM load (one set of five repetitions), consisting of lengthening action (2 s) and shortening action (2 s), with the aid of a metronome. The knee joint angle was measured using an electronic goniometer (SG150, Biometrics, UK). Parallel squat depth was defined in advance at the position at which the thigh was parallel to the floor. The depth was controlled using a tense rope set at the height of each participant’s squat depth and feedback was provided by an examiner throughout the exercises. As a result, a two-way analysis of variance (ANOVA) indicated that there was no main effect of time (before and after the intervention, *P* = 0.250) or condition (VL, BF and CON conditions, *P* = 0.570) or interaction of the two factors (*P* = 0.162) on knee joint angle at the depth position. This suggests that knee and hip joint kinematics during the squat exercise was almost matched throughout the experiment by controlling of the standing position and squat depth. The determination of 10 RM load of the parallel squat was performed after several submaximal squat exercises at light-to-moderate load as a warm-up. The load was then increased until the participant could successfully lower and raise the bar from a sitting position in which the thigh was parallel with the floor 10 times but failed to achieve an 11th repetition. The participant determined his 10 RM (59 ± 10 kg) with sufficient rest within four attempts.

### Data analysis

The EMG, torque, force of vibration and knee joint angle data were simultaneously recorded at 1 kHz sampling frequency and stored in a personal computer after A/D conversion (PowerLab16/35, ADInstruments, Australia). In the strength measurements before the intervention, data during the two trials were averaged and used for further analyses. The root mean square values of EMG signals (RMS-EMGs) were calculated over a 0.5 s period around the peak torque. For the parallel squat, the RMS-EMG at each repetition was calculated separately in the lengthening and shortening phases, which were determined from the knee joint angle data, and data of five repetitions were averaged in each phase. Each muscle’s RMS-EMG was normalized to that during MVC trials before the intervention. In addition, to examine the inter-muscle difference in the magnitude of muscle activations, the RMS-EMGs were also averaged between lengthening and shortening phases before the interventions.

### Statistical analysis

Data are presented as means ± SDs. The statistical analyses were performed using SPSS version 22 (IBM, USA). A two-way ANOVA with repeated measures was conducted to determine the effects of time (before and after the intervention) and condition (VL, BF and CON conditions) on MVC torques and RMS-EMG during MVC trials in each muscle. The relationship between relative change in RMS-EMG of the vibrated muscle and relative change in MVC torque was tested using Pearson’s product moment correlation coefficient. A two-way ANOVA with repeated measures was used to determine whether normalized RMS-EMGs (means of the lengthening and shortening phases) during the squat before the intervention differed among the muscles (VL, VM, RF, BF and SM) and three conditions. To examine the effects of time, condition and phase (lengthening and shortening), a three-way ANOVA with repeated measures was performed on normalized RMS-EMG during parallel squat exercises in each muscle. When a significant interaction or main effect of time was shown, following ANOVA with the Bonferroni multiple-comparison test was used to examine the differences in variables before and after the intervention in each condition and phase. The significance level was set at *P* < 0.05.

## Results

**Fig. 2:**
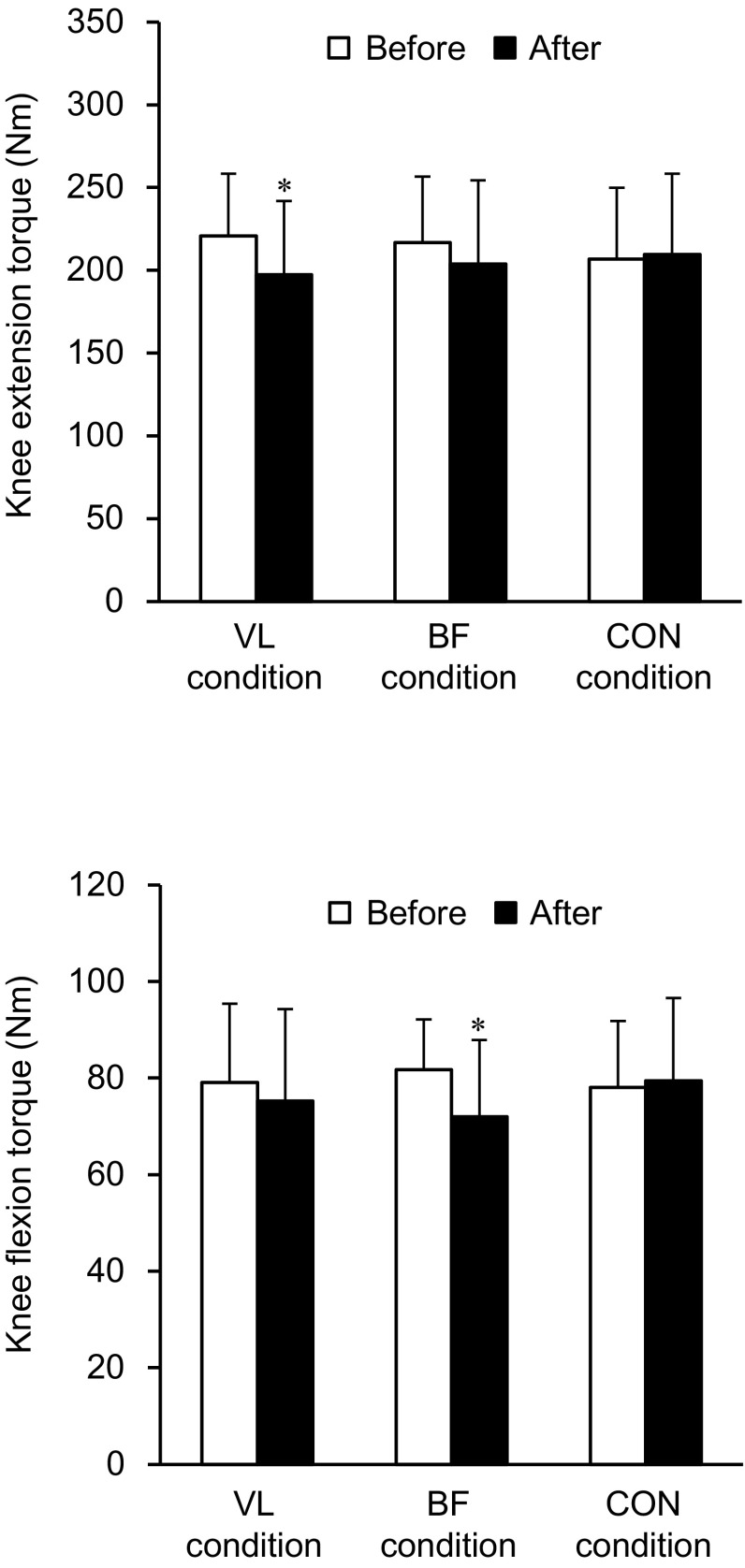
Maximal voluntary isometric contraction torque of the knee extension and knee flexion before and after the intervention. *Asterisk* indicates a significant change after the intervention. VL condition, tonic vibration to the vastus lateralis during quiet sitting for 30 min; BF condition, tonic vibration to the biceps femoris long head during quiet sitting for 30 min; CON condition, quiet sitting for 30 min without muscle vibration. Data are presented as mean ± standard deviation

Figure 2 shows the MVC torques in each condition. There was a significant interaction of time × condition (*P* = 0.011–0.023). No significant differences in baseline measurement of the MVC_KE_ or MVC_KF_ torque were observed among the three conditions (*P* = 0.142–0.559). The MVC_KE_ torque significantly decreased after the intervention in VL condition (*P* = 0.006), whereas it did not change in BF or CON condition (*P* = 0.125–0.607). A decrease in MVC_KF_ torque after the intervention was significant in BF condition (*P* = 0.005) but not in VL or CON condition (*P* = 0.169–0.646).

**Fig. 3:**
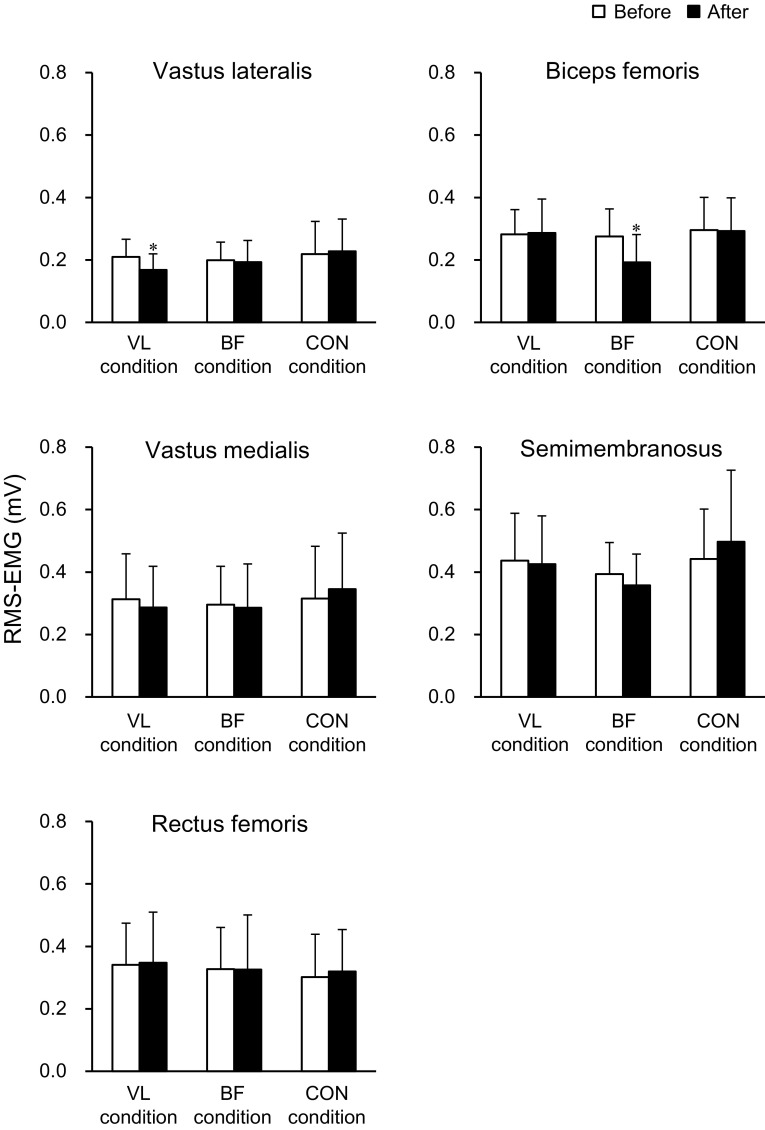
Root mean square value of electromyogram (RMS-EMG) during the maximal voluntary isometric contraction trials before and after the intervention. *Asterisk* indicates a significant change after the intervention. VL condition, tonic vibration to the vastus lateralis during quiet sitting for 30 min; BF condition, tonic vibration to the biceps femoris long head during quiet sitting for 30 min; CON condition, quiet sitting for 30 min without muscle vibration. Data are presented as mean ± standard deviation

The RMS-EMGs during MVC trials are shown in Fig. 3. There was a significant interaction of time × condition for VL (*P* = 0.001) and BF (*P* = 0.017). No significant differences were seen in baseline measurements of either muscle among the three conditions (*P* = 0.424–0.705). The RMS-EMG of VL significantly decreased after the intervention in VL condition (*P* < 0.001), whereas it did not change significantly in the other two conditions (*P* = 0.084–0.625). A decrease in RMS-EMG of BF was significant in BF condition (*P* < 0.001) but not in VL or CON condition (*P* = 0.875–1.000). In contrast, no significant main effects or interaction were observed in RMS-EMG of VM, RF or SM (*P* = 0.071–0.883). The relative change in RMS-EMG of VL (*r* = 0.640, *P* = 0.014) or BF (*r* = 0.662, *P* = 0.010) was correlated with the relative change in MVC_KE_ or MVC_KF_ torque in VL or BF condition, respectively.

**Fig. 4:**
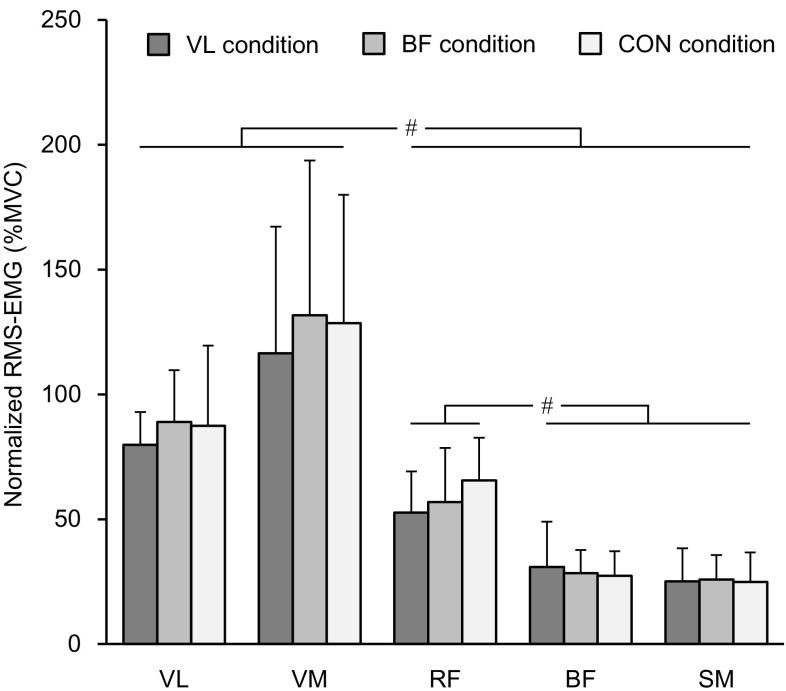
Root mean square value of electromyogram (RMS-EMG) during parallel squat before the intervention (mean of the lengthening and shortening phases) relative to those recorded at maximal voluntary isometric contraction (MVC). *Number sign* indicates a significant difference between muscles. *VL* vastus lateralis, *VM* vastus medialis, *RF* rectus femoris, *BF* biceps femoris long head, *SM* semimembranosus. VL condition, tonic vibration to the vastus lateralis during quiet sitting for 30 min; BF condition, tonic vibration to the biceps femoris long head during quiet sitting for 30 min; CON condition, quiet sitting for 30 min without muscle vibration. Data are presented as mean ± standard deviation

The normalized RMS-EMGs before the intervention are indicated in Fig. 4. A main effect of muscle (*P* < 0.001) was significant without a significant main effect of condition (*P* = 0.280) or interaction of the two factors (*P* = 0.571). The normalized RMS-EMGs of VL and VM were significantly greater than those of RF, BF and SM (*P* ≤ 0.001–0.005), and that of RF was significantly greater than those of BF (*P* < 0.001) and SM (*P* < 0.001).

**Fig. 5:**
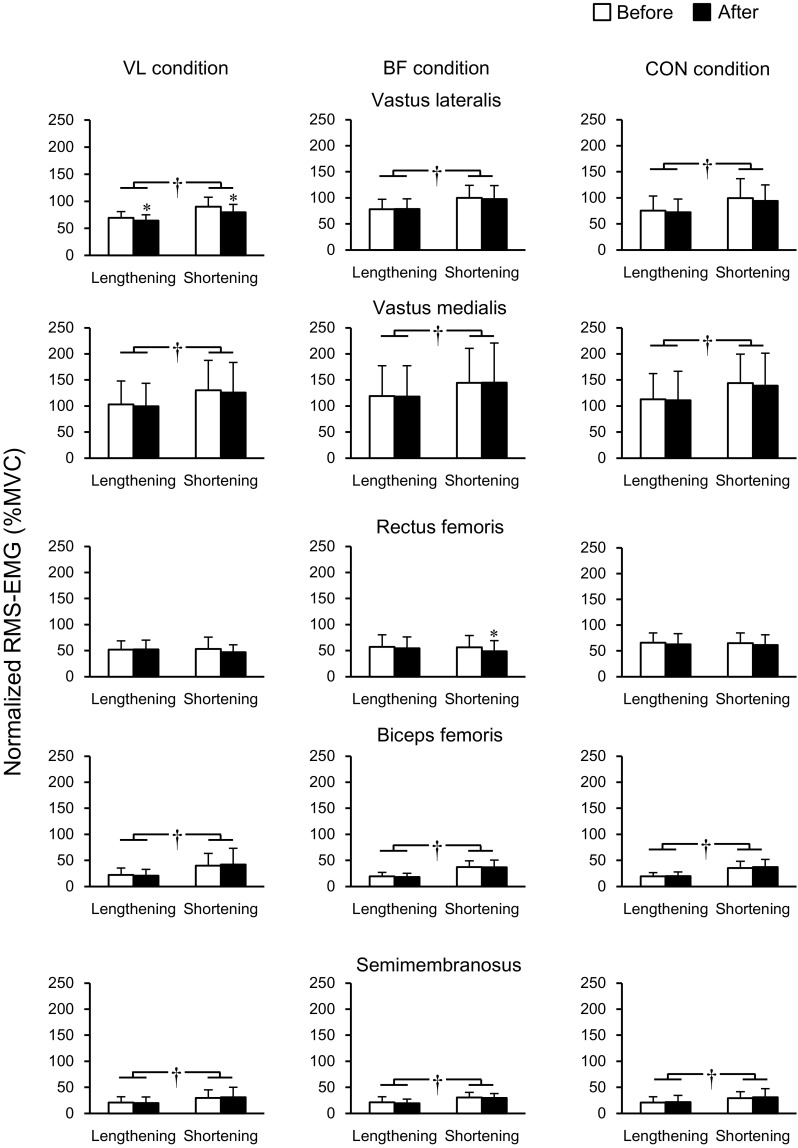
Root mean square value of electromyogram (RMS-EMG) relative to those recorded at maximal voluntary isometric contraction (MVC) during parallel squat at lengthening and shortening phases before and after the intervention. *Asterisk* indicates a significant change after the intervention. *Dagger symbol* denotes a significant difference between the lengthening and shortening phases. VL condition, tonic vibration to the vastus lateralis during quiet sitting for 30 min; BF condition, tonic vibration to the biceps femoris long head during quiet sitting for 30 min; CON condition, quiet sitting for 30 min without muscle vibration. Data are presented as mean ± standard deviation

Data of normalized RMS-EMGs at lengthening and shortening phases during squat exercises are indicated in Fig. 5. A significant main effect of time was seen on RMS-EMG of VL (*P* = 0.005) and RF (*P* = 0.004) but not in the other three muscles (*P* = 0.299–0.880). No baseline differences were seen in any muscles or phases among the three conditions (*P* = 0.109–1.000). Follow-up analyses demonstrated that the RMS-EMG of VL decreased significantly in VL condition (*P* < 0.001 in both lengthening and shortening phases) without significant changes in either BF (*P* = 0.435–0.807) or CON (*P* = 0.153–0.186) condition. The RMS-EMG of RF was reduced significantly at the shortening phase in BF condition (*P* = 0.002), whereas no changes were observed in other conditions (*P* = 0.074–0.916). Except for RF (*P* = 0.607), a main effect of phase was significant (*P* < 0.001 in all muscles except for RF). The normalized RMS-EMGs were significantly greater in the shortening than in the lengthening phase.

## Discussion

The present study demonstrated that prolonged vibration to the agonist muscle decreased maximal voluntary isometric strength and its activation. These results are consistent with previous studies (Jackson and Turner 2003; Kouzaki et al. 2000; Shinohara et al. 2005). Moreover, magnitude of reduction of the vibrated muscles’ activation was associated with the corresponding decrease of the isometric strength. These findings suggest that neural inputs to α motoneurons originating from Ia afferent fibers were suppressed by the 30 min muscle vibration. Contrary to our hypothesis, however, prolonged vibration to the synergist (VL) or antagonist (BF) muscle did not increase RF activation during squat exercises, with the inter-muscle difference in the muscle activation (vasti > RF). It is likely, therefore, that lower activation of the biarticular RF compared with monoarticular VL and VM during multi-joint leg extensions does not result from Ia afferent-mediated inhibitory connections between synergistic muscles and/or between agonist and antagonist muscles.

Consistent with previous findings (Escamilla et al. 1998, 2001; Ploutz-Snyder et al. 1995), the magnitude of muscle activation of RF during squat exercise was lower than those of the vasti (Fig. 4). In a previous study (Ema et al. 2016a), no differences were observed in the magnitude of muscle activation between RF and the vasti during single-joint knee extensions, and the vasti activations were similar between single-joint knee extensions and multi-joint leg extensions at the same exercise intensities. Therefore, there would have been two possibilities in the current study: less excitation and more inhibition of RF motoneurons compared with the vasti during multi-joint leg extensions. Regarding the less excitation mechanisms, there remains a possibility of less facilitatory input to RF motoneurons compared with the vasti during multi-joint leg extensions. To clarify this point, we would need to evaluate motor cortex excitability during MVC and squat using technique such as transcranial magnetic stimulation; however, the evaluation is practically difficult. The inhibitory mechanisms still remain in the present findings. As mentioned above, inhibitory input to RF motoneurons from the synergist and antagonist muscles through Ia afferent-mediated connections has less impact on the lower RF activation during multi-joint leg extensions. Although only VL and BF were vibrated to examine the effect of Ia afferent-mediated inhibitory connections to RF from the synergistic and antagonistic muscles, respectively, in the current study, it is not reasonable to suppose that synergistic or antagonist muscles other than VL or BF inhibited RF motoneurons. In contrast, the excitability of Ia inhibitory interneurons is controlled by factors such as corticospinal descending inputs (Nielsen et al. 1993) and Renshaw cells (Hultborn et al. 1971) as well as Ia afferents. Therefore, we cannot completely exclude the inhibitory mechanisms in RF activation. Indeed, the estimated corticospinal descending inputs to Ia inhibitory interneurons were related to inter-individual variability of the magnitude of reciprocal Ia inhibition at the ankle joint (Kubota et al. 2014). Moreover, inhibitory mechanisms between agonist and antagonist muscles are reported to be differently organized between hinge and ball joints (Wargon et al. 2006). The reported difference complicates interpretation of the present findings, because the biarticular RF and hamstring muscles cross both hinge (knee) and ball (hip) joints. Taken together, although it is difficult to identify the mechanisms underpinning lower activation of RF than the vasti during multi-joint leg extensions, peripheral inhibitory input from Ia afferents originating from synergist and/or antagonist muscles, which has been proposed as the factor of unique activation of RF during multi-joint leg extensions (Ema et al. 2016a; Yamashita 1988), seems unlikely.

The muscle activation of VL during squat exercise as well as during MVC_KE_ trials decreased after prolonged VL vibration. To the best of our knowledge, this is the first study showing a significant effect of prolonged muscle vibration on the muscle activation during multi-joint dynamic as well as single-joint isometric contractions. We failed to find increases in muscle activation in other muscles involving RF to compensate for VL activation attenuation, but this might have occurred in some muscles that were not investigated (e.g., vastus intermedius, semitendinosus, and gluteus maximus). In contrast, the corresponding decrease in BF activation during MVC_KF_ was not followed by that during squat after prolonged BF vibration. A previous study observed that a decline of discharge rate induced by prolonged vibration was more prominent in high-threshold than low-threshold motor units during MVC trials (Bongiovanni et al. 1990), suggesting that Ia afferent activity plays an important role in recruiting high-threshold motor units during contractions. It was shown that the proportion of type II fibers of BF was lower than those of VL (Johnson et al. 1973). In the present study, the extent of muscle activation during squat exercises was lower in BF than VL (Fig. 4), and the magnitude of BF activation was approximately 30% of that during MVC trials. During submaximal shortening and lengthening contractions, the recruitment order of motor units is similar between the two contractions (Pasquet et al. 2006) and consistent with the size principle (Duchateau and Enoka 2016). It is thus possible that the recruitment of high-threshold motor units was insufficient in BF during squat exercises, resulting in the lack of response in BF activation following prolonged BF vibration. Another possible explanation is task dependency of the effect of prolonged vibration. We measured MVC_KF_ torque but not hip extension MVC torque before and after the interventions. It has been shown that muscle activation of the biarticular RF was lower during hip flexions than knee extensions (Miyamoto et al. 2012; Watanabe et al. 2012). If a corresponding difference is occurred in BF (i.e., lower activation during hip extensions than knee flexions), MVC torque of the hip extension might be less affected by prolonged muscle vibration.

There are some limitations to the present study. First, the use of untrained participants may have resulted in the large variability in the muscle activations and joint kinematics during squat. Therefore, we may have failed to find significant changes in RF activation and/or compensations for decreased VL activation during squat. Second, vibration-induced decrement in RMS-EMG of VL and BF might be due to changes in peripheral factors rather than regulation of the neural command. However, prolonged patellar tendon vibration did not affect M-wave amplitudes of VL and/or VM (Fry and Folland 2014; Saito et al. 2016), suggesting that the above possibility was unlikely in the present study. Finally, the smaller RMS-EMG of RF compared with the vasti during squat might be related to the inter-muscle difference in the muscle shortening during the exercise. Higher muscle shortening velocity during concentric MVC resulted in larger EMG amplitudes (Babault et al. 2003). This may partly explain the greater normalized activations of VL and VM than RF during squat considering the possibility of smaller muscle length change in RF because of its biarticular nature. In contrast, a larger amount of muscle shortening during the shortening phase of squat can result in the smaller EMG amplitudes because the number of activated muscle fibers within the recording volume of the surface electrodes could decrease with muscle shortening. If this point is critical in the current study and neural command during squat is modulated similarly between the vasti and RF, normalized muscle activations would be smaller in the vasti than in RF. This assumption is not consistent with the present results. Taken together, it is likely that lower EMG amplitude of RF compared with the vasti during squat is a result of neural command modulation.

In conclusion, the current study revealed that muscle activation of the biarticular RF during squat exercises did not increase after prolonged VL or BF vibration for 30 min. Consistent with previous studies, the magnitudes of muscle activation during squat exercises were lower in the biarticular RF than monoarticular vasti muscles. The present findings suggest that difference in neuromuscular activation among the synergistic muscles during multi-joint exercises is not a result of modulation by Ia afferent-mediated inhibitory connections between synergistic muscles and/or between agonist and antagonist muscles; other mechanisms are involved at the supraspinal and spinal levels.

## Abbreviations

ANOVA: Analysis of variance
BF: Biceps femoris long head
EMG: Electromyography
MVC_KE_: Maximal voluntary isometric contraction of knee extensors
MVC_KF_: Maximal voluntary isometric contraction of knee flexors
RF: Rectus femoris
RM: Repetition maximum
RMS-EMG: Root mean square of electromyographic amplitude
SM: Semimembranosus
VL: Vastus lateralis
VM: Vastus medialis
